# Large-Scale Fabrication of Photonic Nanojet Array via Template-Assisted Self-Assembly

**DOI:** 10.3390/mi11050473

**Published:** 2020-04-30

**Authors:** Pengcheng Zhang, Xi Chen, Hui Yang

**Affiliations:** 1 Laboratory of Biomedical Microsystems and Nano Devices, Bionic Sensing and Intelligence Center, Institute of Biomedical and Health Engineering, Shenzhen Institutes of Advanced Technology, Chinese Academy of Science, Shenzhen 518055, China; pc.zhang@siat.ac.cn (P.Z.); xi.chen@siat.ac.cn (X.C.); 2 CAS Key Laboratory of Health Informatics, Shenzhen Institutes of Advanced Technology, Chinese Academy of Sciences, Shenzhen 518055, China

**Keywords:** photonic nanojet, photonic nanojet array, self-assembly, template-assisted self-assembly, patterning efficiency

## Abstract

A large-scale homogenized photonic nanojet array with defined pattern and spacing facilitates practical applications in super-resolution imaging, subwavelength-resolution nanopatterning, nano objects trapping and detection technology. In this paper, we present the fabrication of a large-scale photonic nanojet array via the template-assisted self-assembly (TASA) approach. Templates of two-dimensional (2D) large-scale microwell array with defined pattern and spacing are fabricated. Melamine microspheres with excellent size uniformity are utilized to pattern on the template. It is found that microwells can be filled at a yield up to 95%. These arrayed microspheres on the template serve as microlenses and can be excited to generate large-scale photonic nanojets. The uniformly-sized melamine spheres are beneficial for the generation of a homogenized photonic nanojet array. The intensity of the photonic nanojets in water is as high as ~2 fold the background light signal. Our work shows a simple, robust, and fast means for the fabrication of a large-scale homogenized photonic nanojet array.

## 1. Introduction

The photonic nanojet (PNJ) is an electromagnetic beam of high intensity that is generated by normal plane wave incidence on a dielectric microcylinder or microsphere of diameter greater than the illuminating wavelength [1,2]. These nanojets have a narrow beam waist smaller than the diffraction limit and propagate with little divergence for several wavelengths. Owning to its high intensity, narrow waist and the enhanced backscattering effect with the nanoparticles, the PNJ has been widely used in super-resolution imaging [3,4,5,6,7], fluorescence enhancement [8,9,10,11], enhanced Raman scattering [12], subwavelength resolution nanopatterning [13,14], nano-objects or molecules trapping and detection [15,16,17,18,19,20,21,22], and so on. In the work cited above, PNJs were mainly generated either by an individual dielectric microsphere, randomly distributed microspheres or self-assembled microsphere films. When it comes to the practical applications, there is a demand for generating large-scale homogenized PNJs simultaneously with a defined pattern and inter-PNJ spacing, especially in the fields of super-resolution imaging, nanopatterning, nano objects trapping and detection technology. The self-assembled microsphere film can generate PNJs on a large scale. However, the pattern and spacing between adjacent PNJs cannot be tuned or increased, since the microspheres in the film are mainly closely packed into a hexagonal pattern, with no room to adjust. This could be improved by utilizing microspheres with different sizes, aiming to adjust their lattice constant of the film. Nevertheless, the range of achievable PNJ spacing is still quite limited, due to the limited number of spices or sizes of microspheres that are suitable for PNJ generation.

Numerous approaches have been developed to organize nano- or microscopic components into patterns through self-assembly [23]. Some of the approaches focus on programming of the nano- or microscopic components to organize in a predetermined way [24,25], while others tailor the environment to direct and control the assembly [26,27,28,29]. To obtain self-assembly structures with well-defined patterns, utilizing a pre-designed surface on which these nano- or microscopic components will self-assemble and eventually reside would result in a desirable assembly. Template-assisted self-assembly (TASA) is such an approach that is used to fabricate nano- or microsphere arrays with well-controlled spatial patterns on surfaces patterned with two-dimensional arrays of templates [30]. In this approach, a template with pre-designed pattern is utilized as the substrate for the self-assembly process. Nano- or microscopic components in the patterning liquid are transited over the substrate to dewet through the fine structures on the template. The components in the liquid are pushed into the physical template to form a densely packed structure. As a result, self-assembly structures with a well-defined pattern can be obtained. Here in this work, we report on the fabrication of a large-scale photonic nanojet array using the TASA approach. A microwell template of a 2D large-scale array was fabricated using conventional microfabrication technology. Melamine microspheres were then assembled on these templates. By controlling the parameters during the TASA process, up to 95% of the loading efficiency can be achieved. These well-ordered arrays of microsphere show excellent optical focusing capability. This approach provides a simple, robust, and fast means for the fabrication of a large-scale homogenized photonic nanojet array and, in future, it may develop into versatile tools to image or detect nanometric objects of biological or environmental importance.

## 2. Materials and Methods 

### 2.1. Materials

Carboxyl-functionalized melamine microspheres (3 μm in diameter, standard deviation <0.1 μm) were purchased from Sigma-Aldrich (St. Louis, MO, USA). Hydrophobic polymer of carbon–fluorine (Cytop-809M) was purchased from AGC (Tokyo, Japan). pH buffer solution was purchased from Aladdin (Shanghai, China). Photoresist (AZ4620) was purchased from Merck (Darmstadt, Germany). AZ 300MIF developer was purchased from Integrated Micro Materials (Argyle, Texas, USA). Float glass wafer (Ø4 in., 550 μm in thickness) was used for microfabrication.

### 2.2. Methods

#### 2.2.1. Fabrication of the Microwell Array Template

**Figure 1:**
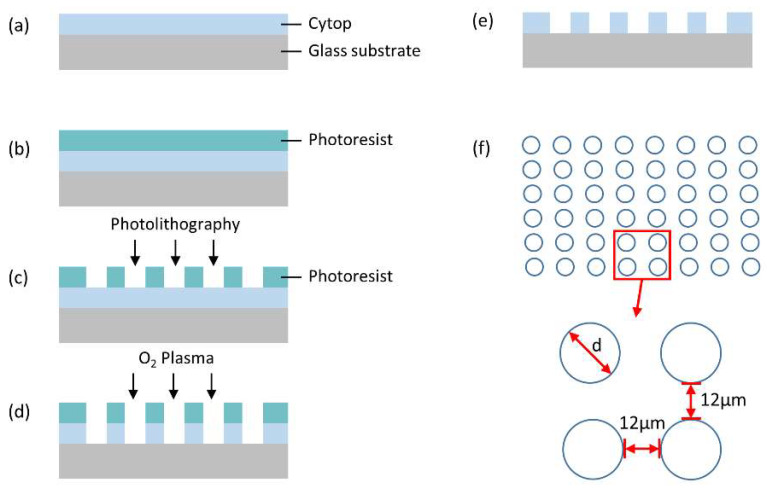
Schematic illustration of the microfabrication process and the design of the microwell array template. (**a**) Cytop is coated on the top of the glass wafer. (**b**) Photoresist is spin-coated on the Cytop layer. (**c**) The glass wafer is patterned by standard photolithography. (**d**) The photoresist layer and Cytop layer are etched by O_2_ reactive ion etching. (**e**) Photoresist layer is removed and the microwell array template is obtained. (**f**) The microwell template is arranged in a quadrilateral pattern with separations of 12 µm in both x- and y- directions. Three different sizes of the microwell are designed (d = 2.4 µm, 3.3 µm and 4.3 µm, respectively).

The microwell array template was fabricated using standard microfabrication technology. The main fabrication process of the templates is schematically illustrated in Figure 1a–e. The microwell array template was designed with an area of ~15 mm × 8.6 mm containing 0.17 billion microwells with three different sizes (2.4 µm, 3.3 µm and 4.3 µm in diameter). The microwells were arranged in a quadrilateral pattern with separations of 12 µm in both x- and y- directions, Figure 1f. For the fabrication process, a glass wafer was cleaned by ethanol and isopropanol twice and dried under nitrogen. Then it was exposed to an oxygen plasma to increase its affinity to the Cytop layer. A layer of Cytop–809M (2.5 µm thickness) was spin-coated on the glass wafer. Then the glass wafer with the Cytop on surface was heated on a hot plate to cure the Cytop layer, by elevating the temperature from room temperature to 180 °C in a gradient. To completely volatilize the solvent in the Cytop and to improve adhesion with the substrate, it is recommended to perform final baking at as high a temperature as possible (180 °C in our case). Afterwards, the photoresist AZ 4620 (8 µm thickness) was coated on the Cytop layer. Then the glass wafer was patterned by standard photolithography and followed by O_2_ reactive ion etching (RIE). After etching, the photoresist layer was then removed and the glass wafer was diced into small chips that were ready to be used for microsphere patterning. 

#### 2.2.2. Patterning of the Melamine Microspheres

**Figure 2:**
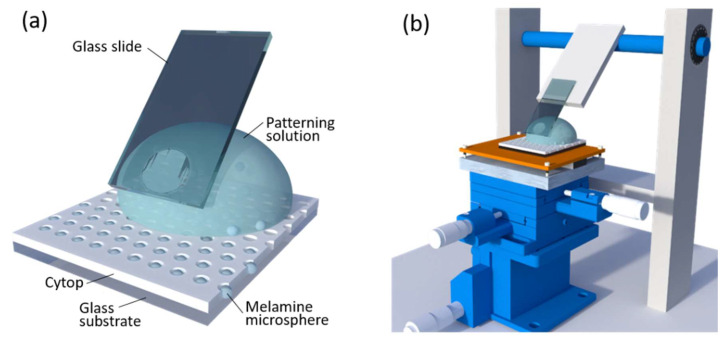
Schematic illustration of (**a**) patterning of the melamine microspheres on the microwell array template by (**b**) adjustable self-assembly setup.

The carboxyl-functionalized melamine microspheres with 3 μm in diameter were used to be patterned on the microwell array template (Figure 2a). To study the pH dependent patterning efficiency, the microspheres with concentration of 10% solids in aqueous solution was 10× diluted in a buffer with different pH values (pH = 1.0, 3.0, 5.0 and 7.0), respectively, as the patterning solution. A homemade self-assembly setup was used to pattern the melamine spheres on the template via template-assisted self-assembly (TASA) (Figure 2b). The self-assembly setup consists of a rectangular glass slide inclined at an angle of 40°. The glass slide is over a temperature-regulated horizontal copper plate which serves as a support for the microwell array template. The copper plate was fixed on a three-dimensional precision displacement platform. All the patterning process was carried out in an ambient environment with a surrounding temperature of 22 °C and a humidity of ~65% relative humidity (RH). The copper plate was controlled at 24 °C during the TASA process. During the TASA process, 10 μL of the patterning solution was introduced into the wedge that was formed between the glass slide and the template. The template was translated under the patterning solution for 15 patterning cycles (1 cycle = 1 passage of the patterning solution over the array) at a speed ~200 μm/s. After the patterning process, excess microspheres on the array surface were gently washed away by Milli-Q water and the surface was dried by nitrogen. 

#### 2.2.3. Observation of the Photonic Nanojet (PNJ)

**Figure 3:**
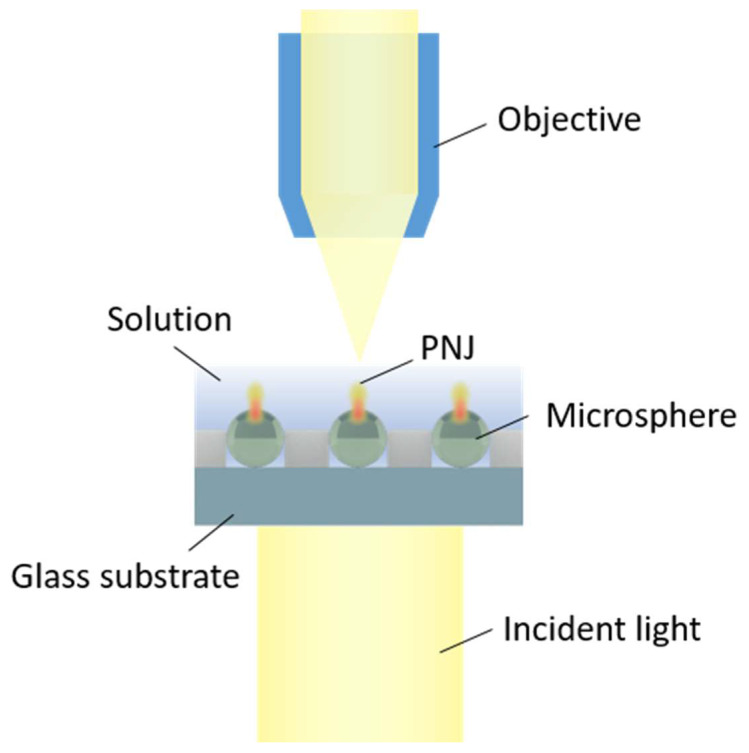
Schematic illustration of the optical setup used in photonic nanojet (PNJ) observation and measurements.

The as prepared microsphere array was configured with a glass ring on the top. Milli-Q water was introduced into the glass ring, in which the microspheres were fully immersed. The microsphere array was illuminated by a light-emitting diode (LED) white light source from the bottom of the glass substrate. A 20× objective with NA of 0.8 (Zeiss Objective Plan-Apochromat, Zeiss, Oberkochen, Germany) was utilized to image the PNJ from the top of the microsphere array, focusing on the focal plane of the microspheres (Figure 3).

## 3. Results and Discussion

**Figure 4:**
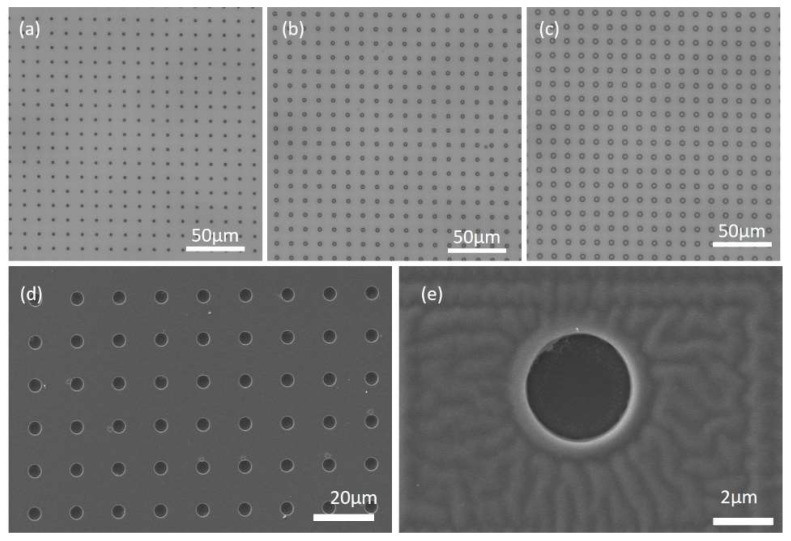
Morphology of the microwells. (**a**–**c**) represent the microwells with the size of 2.4 µm, 3.3 µm and 4.3 µm, respectively, characterized by a conventional optical microscope; (**d**) scanning electron microscope (SEM) image of the 3.3 µm microwells and (**e**) the enlarged SEM image of a single microwell.

The morphology of the fabricated microwells with 2.4 µm, 3.3 µm and 4.3 µm in diameter was characterized by a light microscope and a scanning electron microscope (Zeiss Gemini scanning electron microscope (SEM), Zeiss, Oberkochen, Germany) (Figure 4). It is shown that the fabricated Cytop microwells possess uniform size and well-defined spacing in the same batch. The depth of the microwell is ~2.5 µm measured by a profile meter. Water contact angle was measured as 96.9 ± 0.57 °C on the area which contains the microwell array.

**Figure 5:**
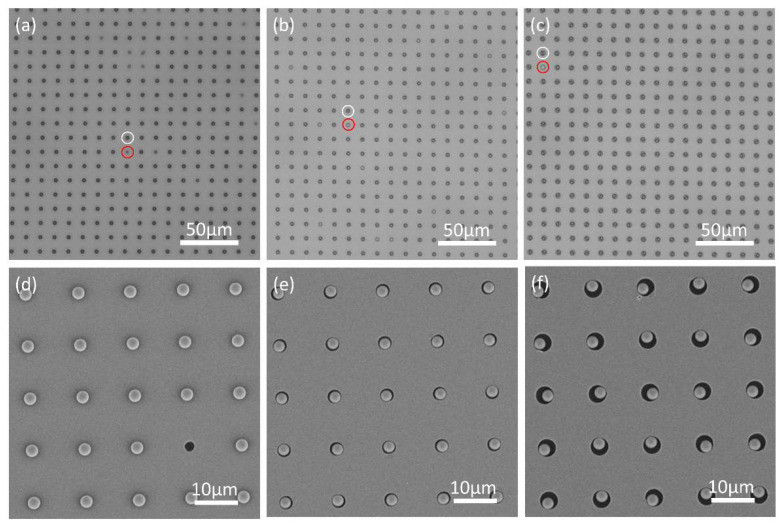
Microsphere array patterned on microwell array template of different sizes. (**a**–**c**) represent the microscopic image of the microspheres loaded on the microwells with the size of 2.4 µm, 3.3 µm and 4.3 µm, respectively. The white circle on each figure indicates the microwell is occupied with a microsphere and the red circle indicates the microwell without microsphere occupation. (**d**–**f**) represent the scanning electron microscope (SEM) images of the microspheres loaded on the microwells with the size of 2.4 µm, 3.3 µm and 4.3 µm, respectively.

After the fabrication of the microwell array, carboxyl-functionalized melamine microspheres of 3 μm were patterned via TASA approach. These commercial available melamine microspheres are practically uniform in size with standard deviation <0.1 μm. Furthermore, the previous work shows that the melamine sphere of 3 μm possesses excellent optical focusing capability than that of other sizes [11], which is beneficial to generate PNJ with best optical focusing capability in our experiment. After the patterning process, the microwell array with the patterned melamine spheres was characterized under an optical microscope and a scanning electron microscope (Figure 5). It is shown that the microspheres are precisely loaded into each individual microwell without access microspheres on the raised regions of the template. Moreover, the configuration of microspheres occupying a microwell is determined by the size of the template microwell and the size of the microspheres. For the microwells with 2.4 µm (Figure 5a), since the size of the microwell is smaller than the microsphere (3 µm), the microspheres were trapped partially on their lower surface into the microwell. This can be seen clearly on the SEM images (Figure 5d). However, for the microwells of 3.3 µm or 4.3 µm, who have larger size than the microsphere, fully entry of the microsphere into the microwell occurred. This can also be observed clearly on the template microwells of 3.3 µm and 4.3 µm (Figure 5e,f), in which the microspheres were pushed onto the sidewall, leaving excess rooms in the microwell.

Furthermore, the microwell with or without melamine microsphere loading can be easily distinguished, as they exhibited different shades and thicknesses on their contours. This allows us to statistically analyze the quality of the patterning, by counting the number of the microwells with or without microsphere. The patterning efficiency η, which indicates the quality of the patterning, is defined as $\eta =N_{load}/N_{tot}$, where $N_{load}$ is the number of microwells containing the melamine microsphere and $N_{tot}$ is the total number of microwells. It is found that the patterning efficiency η was influenced by the number of the patterning cycles, the pH value of the patterning solution, and also the size of the microwell in our experiments. For the approach of TASA we used in this work, more patterning cycles increase the probability that the microspheres encounter the microwells, thus increasing the patterning efficiency. It is found that the patterning efficiency varies from sample to sample with a relative large deviation when counted individually at the end of each patterning cycle. However, a very high efficiency (>95%) can be achieved after 15 patterning cycles for each sample in a patterning solution with proper pH condition. In our experiment, all the samples were conducted and statistically analyzed after 15 patterning cycles.

**Figure 6:**
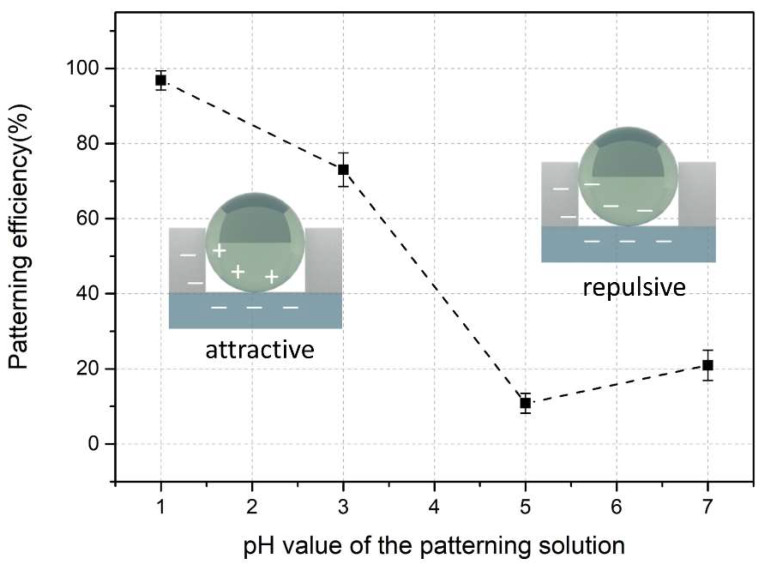
The patterning efficiency of the template with microwells of 4.3 µm versus the pH value of the patterning solution. Sketches illustrate the surface charges of the microspheres and bottom substrate during the patterning process at different pH conditions, indicating the electrostatic charges involved in the patterning process.

The pH value of the patterning solution exhibits a significant influence on the patterning efficiency. To study the pH effect, a patterning solution with different pH values (pH = 1.0, 3.0, 5.0 and 7.0) was used during the patterning process, respectively. We analyzed the patterning efficiency after 15 patterning cycles and the pH dependent patterning efficiency of the template with microwells of 4.3 µm is shown in Figure 6. It is shown that the patterning efficiency strongly depended on the pH value of the patterning solution. With the increase of the pH value, the patterning efficiency decreases. The patterning solution with pH value of 1.0 yielded the highest patterning efficiency (~99%). This result revealed the electrostatic nature of the patterning process. As reported from the literature [22], the pH value of the patterning solution influences the species of the charge on the surface of the microspheres and the bottom substrate, respectively, due to the isoelectric point of different materials. Thus, the electrostatic force caused by these charges has an impact on the entry of the microspheres into microwells. In our experiments, higher pattering efficiencies were obtained for pattering solution with lower pH (≤3.0). Considering the isoelectric point of the melamine microsphere (~3), the Cytop layer (~2) and glass substrate (<3), at a patterning solution of pH lower than 3.0, the microspheres actually hold the positive charges, while the substrate holds negative charges. In this case, their electrostatic interaction is positive, that is, microspheres are attracted into the microwells to a greater extent, thus promoting the patterning efficiency and stability during the patterning process.

Under the optimized patterning cycle and pH value of the patterning solution, the patterning efficiency is >95% for the templates with microwells of 2.4 µm or 3.3 µm, and >99% for the templates with microwells of 4.3 µm. This slight increase of the patterning efficiency (~4%) on templates with larger size of the microwell may arise from the different configurations of microsphere occupying a microwell. In the TASA approach, the size of the microwell influences the configuration and stability of an individual microsphere in the microwell [30], as shown in Figure 5. A microwell with larger size allows the fully entry of the microsphere, thus the electrostatic attraction applies on a larger surface area, providing stronger attraction force and a more stable accommodation. Moreover, the size of the microwell determines the probability of these microspheres encountering the microwells during the patterning process, since an array with larger size of the microwell provides more capture area (interior of the microwell), thus higher chances for the microspheres to be captured. Therefore, an array with microwells of relatively large size would benefit a higher patterning efficiency, although this effect only exhibited a minor influence on the patterning efficiency in our experiment. It should be noted that the data of patterning efficiency in our experiments was collected after 15 patterning cycles. The patterning efficiency can be further increased by additional patterning cycles.

**Figure 7:**
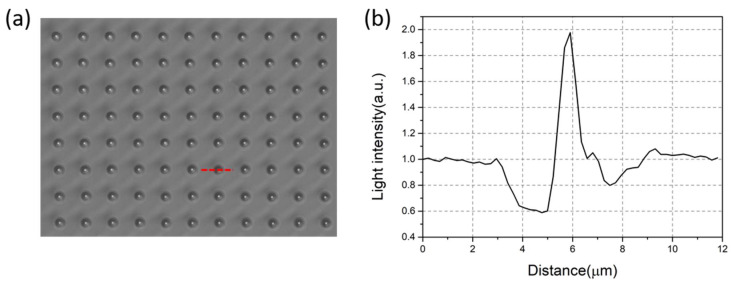
Microlens array and its light focusing property. (**a**) Microscopic image of the microspheres in water solution (refractive index = 1.33) illuminated by the white light from the bottom of the glass substrate. The objective is focused on the top plane of the microspheres where highest light intensity is observed. The light intensity of the dashed red line is presented in (**b**). The peak value of the light intensity at ~6 µm is ~2 times higher than the background signal from the area without microsphere.

To observe the light-focusing effect of the microsphere, the microsphere array was immersed in water solution and illuminated from the bottom of the glass substrate, as illustrated in Figure 3. Under such an optical configuration, the as patterned melamine microspheres served as microlens array and could generate PNJs in the close vicinity of the top surface, which can be directly observed by the objective. Since the PNJs possess high light intensity and narrow waist, they can be observed by first focusing on the vicinity of the top surface of the microsphere, where bright white spots emerged in the view, and then followed by fine tuning the focal plane of the white spots to the highest light intensity, as in Figure 7a. It is shown that the arrayed microspheres showed excellent light focusing capability. Individual microspheres focused the light illumined from the bottom into a highly localized white spot, i.e., the PNJ. The PNJ possessed high light intensity and a peak at the center of the microsphere (Figure 7b). The peak value can be as high as ~2 fold the background light signal. It is demonstrated in Figure 7a that a large-scale PNJ array with a defined pattern and spacing was achieved. Furthermore, the arrayed melamine microspheres possess excellent size uniformity and highly consistent radial position (direction along the incident light) in the microwells. Furthermore, they possess high refractive index (1.68), low light absorbance, and optimal light-focusing capability in water [22]. Thus with these advantages, the PNJs generated by these microspheres in Figure 7a could exhibit nearly identical geometry parameters and height on the radial position. These homogenized PNJ array has great potential in practical applications, especially in super-resolution imaging, subwavelength-resolution nanopatterning, and nano objects trapping and detection.

## 4. Conclusions

A large-scale photonic nanojet array was fabricated via a template-assisted self-assembly approach. Templates of large-scale microwell array of three different sizes were fabricated using conventional microfabrication technology. Uniform-sized melamine microspheres of 3 µm were utilized to assemble on the templates. It is shown that the patterning efficiency is strongly depended on the pH values of the patterning solution. Under the optimized condition, the patterning efficiency can be up to 95% for the templates with different sizes. The melamine microspheres serve as the dielectric microsphere lenses and can generate homogenized PNJs over a large-scale sample surface. The intensity of the PNJs in water can be as high as ~2 fold the background light signal. This TASA approach offers a simple, robust, and fast means for the fabrication of large-scale homogenized photonic nanojet array. Therefore, we believe that our technique that exploits the properties of a large-scale PNJ array could evolve into a versatile tool for super resolution imaging and nano-object detection.
